# Use of Physiotherapy Prior to Total Knee Arthroplasty—Results of the Prospective FInGK Study

**DOI:** 10.3390/healthcare10020407

**Published:** 2022-02-21

**Authors:** Hannes Jacobs, Falk Hoffmann, Djordje Lazovic, Uwe Maus, Gesine H. Seeber

**Affiliations:** 1 Department of Health Services Research, Carl von Ossietzky University Oldenburg, 26129 Oldenburg, Germany; falk.hoffmann@uni-oldenburg.de; 2 University Hospital for Orthopaedics and Trauma Surgery Pius-Hospital, Medical Campus University of Oldenburg, 26121 Oldenburg, Germany; djordje.lazovic@uni-oldenburg.de (D.L.); gesine.seeber@uni-oldenburg.de (G.H.S.); 3 Department of Orthopaedic & Trauma Surgery, University Hospital of Düsseldorf, 40225 Düsseldorf, Germany; uwe.maus@med.uni-duesseldorf.de; 4 Department of Orthopedics, University Medical Center Groningen, University of Groningen, 9713 GZ Groningen, The Netherlands

**Keywords:** physical therapy, osteoarthritis, disease burden, health services research, knee arthroplasty, socioeconomic status

## Abstract

Background: Data regarding physiotherapy (PT) utilization prior to total knee arthroplasty (TKA) are insufficient. Therefore, this study aims to examine which percentage of patients receive PT within 12 months prior to TKA and which factors are associated with its use. Methods: Consecutive patients (≥18 years) undergoing primary or revision TKA in a German university hospital were recruited. A questionnaire including information on PT utilization, demography, and socioeconomics was collected one day prior to surgery and linked to medical hospital records. Multivariable logistic regression was conducted to determine variables associated with the use of PT. Results: A total of 241 out of 283 (85%) patients participated (60% female; mean age: 68.4 years). Overall, 41% received PT at least once during 12 months prior to TKA, women more frequently than men (48% vs. 29%). Although high disease burden was associated with increased utilization, about one in two in this condition did not receive PT. Multivariable logistic regression showed that age 75+ years, low education level, and moderate-to-severe depressive symptoms were associated with decreased PT utilization. Conclusions: We found low use of recommended PT management in patients prior to TKA. This potential underuse was even higher in some vulnerable subgroups, indicating inequalities. Prescribers as well as patients should integrate PT more consistently into osteoarthritis management.

## 1. Introduction

Osteoarthritis (OA) is one of the most prevalent chronic joint diseases and can lead to a considerably diminished quality of life (QoL) [1]. It is associated with increased age, and its prevalence is higher in females than in males [2]. The disease is characterized by progressive degenerative changes in the joints, accompanied by increased pain, reduced joint function, as well as muscle weakness and poor proprioception [3,4]. The most commonly affected joint is the knee, which also contributes the most to OA burden [5]. Typically, knee OA management is limited to symptomatic treatment aiming to address the aforementioned symptoms. However, end-stage knee OA often necessitates total knee arthroplasty (TKA), which is recommended by guidelines as a cost-effective final intervention [6]. Notably, up to 15–20% of patients report being dissatisfied following TKA [7]. Those patients perceive only minor benefits and describe an overall poor outcome. Such dissatisfaction accompanied by a limited lifespan of a knee prothesis [8] may lead to high revision rates. Correspondingly, guidelines recommend first and foremost following non-surgical OA management before performing TKA as the final option once patients respond insufficiently to conservative approaches [9,10].

Physiotherapy (PT) has demonstrated major potential in decreasing OA-related pain and maximizing joint function [11,12]. Thus, this measure has been recognized as playing an essential role in non-surgical OA management [13,14]. Guidelines recommend at least 6 months of conservative management, including PT, for patients with symptomatic knee OA prior to surgery [15]. However, data on PT utilization prior TKA are scarce and vary widely across studies [16,17,18,19,20,21]. A United States routine data analysis reported a proportion of only 14% having received PT within the 12 months prior to TKA [16]. In contrast, a similar German routine data analysis observed a higher proportion (49%) of pre-TKA PT utilization [17]. Other available studies only assessing “ever” use of PT prior to TKA found prevalences of 44–73% [18,20,21].

Few of the aforementioned studies determined factors associated with pre-TKA PT utilization. Most studies report that females receive PT more frequently than males [17,18,19,22]. Findings for age are inconsistent; while Power et al. observed no age-related differences in PT utilization one year prior to TKA [19], King et al. showed lower PT utilization in older TKA patients (18). Higher socioeconomic status (SES) was associated with increased PT utilization 12 months prior to TKA [18,19]. However, only one study conducted a multivariate analysis to evaluate predictors for pre-TKA PT utilization [19]. Furthermore, any potential influence of important clinical and patient-reported factors, such as functional status, psychological well-being, or body mass index (BMI), have not yet been sufficiently evaluated. However, identifying vulnerable subgroups prone to inadequate PT utilization is critical to raise healthcare providers’ and policy makers’ awareness—a prerequisite to establish remedial action.

Therefore, this study aims to assess PT utilization 12 months prior to TKA as well as to examine determinants that are associated with higher PT utilization.

## 2. Materials and Methods

### 2.1. Study Design and Study Population

This prospective cohort study was conducted in a 58-bed orthopedics department of a university hospital in a city of 170,000 residents in northwestern Germany. Patients undergoing unilateral primary or revision TKA were consecutively recruited between December 2019 and May 2021. Patients aged <18 years, those with a malignant tumor and a projected life expectancy <12 months as well as patients with language barriers or cognitive impairment rendering them unable to give informed consent or complete the questionnaire were excluded. According to a priori sample size calculation using OpenEpi (Version 3.01), we planned to include 240 participants. Assuming that 70% will use PT one year following TKA, which was one of the primary outcomes in the FInGK project, a net sample of n = 191 was necessary to estimate an accompanying 95% confidence interval with a precision of ±6.5% (63.5–76.5%). Based on a comparable study [23], we assumed that 80% of TKA patients can be followed up for one year, resulting in our final sample size of 240. 

### 2.2. Data Collection

Data were collected pre-operatively (usually at admission one day prior to surgery) as well as 2 and 12 months after surgery (via postal survey). For this cross-sectional study, analyses combined data from patient self-reported baseline questionnaires and clinical data obtained from hospital records.

#### 2.2.1. Survey Data

The baseline questionnaire was divided into the following sections: (1) utilization of health services, (2) sociodemographic and lifestyle factors, (3) pain and function of the impaired knee, (4) QoL and psychological well-being, and (5) participation restrictions in daily life as well as social relationships and support.

Utilization of PT was determined by asking patients whether they had received PT for the impaired knee joint in the past 12 months. Furthermore, type of PT (including (a) therapeutic exercises, therapeutic massage, manual therapy, or traction therapy, (b) electrotherapy, (c) thermotherapy, (d) manual lymphatic drainage, (e) other) and the number of treatment sessions were evaluated.

Pain and functional status were assessed using the Western Ontario and McMaster Universities Osteoarthritis Index (WOMAC) [24], which has been validated among TKA patients [25]. The WOMAC comprises 24 questions arranged in three subscales considering the components pain, stiffness, and joint function. Each question is scored on a 5-point Likert scale. Each subscale and the three subscales’ total score were summed up (higher scores indicating more pain or disability). In addition, affliction duration of the impaired knee and current use of analgesics were assessed.

The Short Form 36 (SF-36) is a validated and reliable tool for assessing health-related QoL (including the dimension of general health) in patients with musculoskeletal conditions [26]. In accordance with previous investigations [27], subjects’ general health status was assessed by a single SF-36 item [28]. Psychological well-being/presence of depressive symptoms was assessed using the WHO Well-Being Index (WHO-5) [29]. Scores were transformed (0–100) and categorized into “moderate-to-severe” (0–28), “mild” (29–50), and “no” (>50) depressive symptoms as validated in previous studies [29,30]. Patients were also asked whether they are living alone in their household. We divided school education into low, middle, and high levels according to the International Standard Classification of Education (ISCED) [31,32]. In the German school system, a low educational level corresponds to 9 years of schooling or leaving school without having graduated (ISCED level 0–2B), a medium educational level to 10 years of schooling (level 2A), and a high educational level to 12 or 13 years of schooling (level 3A), which includes access to higher education.

#### 2.2.2. Data from Medical Records

Data from hospital medical records included subjects’ age and sex. Body mass index (BMI) was obtained from height and weight as determined one day prior to surgery. Missing values (n = 4) were completed via patient self-report. Primary and secondary diagnoses based on the International Classification of Diseases, Tenth Revision, German Modification (ICD-10-GM) were retrieved, and comorbidity conditions were categorized by the Elixhauser Comorbidity Index. This index was developed for studies using administrative hospitalization databases and includes 31 severe comorbidities [33].

### 2.3. Statistical Analyses

Study population characteristics were analyzed descriptively (percentages, mean, SD) and stratified by sex. The proportion receiving PT was calculated with a 95% CI. Non-overlapping 95% CIs were considered statistically significant.

Univariable logistic regression was used to evaluate characteristics that are associated with PT utilization. We included sex (female, male), age groups (18–64 years, 65–74 years, 75+ years), education levels (low, middle, high), BMI (<25 kg/m^2^, 25–<30 kg/m^2^, ≥30 kg/m^2^), number of comorbidities (0, 1–2, ≥3), WOMAC score (divided into quartiles), affliction duration (<3 years, ≥3–<6 years, ≥6 years), presence of depressive symptoms (WHO-5; no, mild, moderate-to-severe), and use of analgesics (yes, no) as covariates. Finally, all variables were included in a multivariable model. ORs were calculated with a 95% CI and considered statistically significant if the accompanying 95% CI does not include 1.

Data analyses were performed with SAS (Version 9.4, SAS Institute, Cary, NC, USA).

## 3. Results

### 3.1. Response and Baseline Characteristics of the Study Population

**Figure 1:**
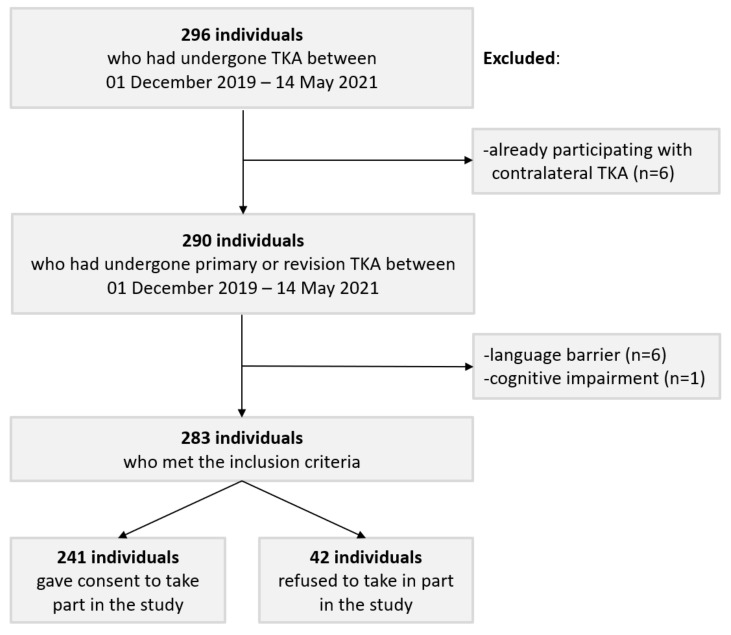
Flowchart of the study population.

After excluding individuals with a language barrier (n = 6), cognitive impairment (n = 1), and those already participating in the study with their contralateral TKA (n = 6), a total of 283 eligible patients underwent primary or revision TKA between 1 December 2019 and 14 May 2021 (Figure 1). Of this group, 241 patients consented to take part in the study, resulting in an 85% response rate. Patients who refused participation were slightly older (mean: 73.9 vs. 68.4 years) and more often female (74% vs. 60%).

Main study population characteristics, stratified by sex, are shown in Table 1. Overall, every second patient had a low educational level and 23% were living alone. The general health status was fair to (very) poor in 77% of patients. Approximately two-thirds were categorized in the BMI group ≥30 kg/m^2^ (mean BMI: 32.9 kg/m^2^), and 36% had ≥3 comorbidities. The mean WOMAC score was 50.5, at an interquartile range (IQR) of 40.0 to 59.0. Afflictions of the impaired knee had lasted more than 6 years in 39% of the study population. Over one-third had moderate-to-severe depressive symptoms, and 71% were currently using analgesics. Male participants more commonly had high educational levels (24% vs. 14%). Female participants, in contrast, had a higher mean WOMAC score (53.2 vs. 46.8) and more commonly used analgesics (81% vs. 58%).

### 3.2. Utilization of Physiotherapy

Overall, 41% of the study population received PT during the 12 months prior to TKA. Therapeutic exercises, therapeutic massage, manual therapy, or traction therapy were prescribed most commonly (for 38% of the study population), followed by manual lymphatic drainage (11%), electrotherapy (4%), thermotherapy (2%), and other types of PT (2%; Supplementary Material S1). The mean number of treatment sessions was 9.6 (IQR: 12–30; Supplementary Material S2).

The proportion of patients who received PT was significantly higher in females than in males (48% vs. 29%; Table 2) for every type of PT (Supplementary Material S1). 

Furthermore, PT use was higher in patients currently using analgesics (yes: 46% vs. no: 25%). The proportion of individuals receiving PT increased from 36% in patients 18–64 years old to 51% in the age group of 65–74 years and decreased again in the age group ≥75 years (32%). PT was received more commonly by patients with a low BMI as well as by patients with high educational level (50%), when compared to those with middle (41%) or low (36%) educational levels. Patients with a higher WOMAC score had increased PT frequencies. While 48% of patients with a score ≥59.0 received PT, only 28% of those with a score <40.0 did. Patients with afflictions of the impaired knee lasting 3 years to <6 years were prescribed PT more frequently (50%) than those who had been afflicted for <3 years or ≥6 years (37% and 34%, respectively). Patients who were not living alone had somewhat lower PT utilization frequencies, as did patients in a fair general state of health or patients with >0 comorbidities. No differences or only minor differences were observed with regard to depressive symptoms.

### 3.3. Univariable and Multivariable Logistic Regression: Predictors for Utilization of Physiotherapy Prior to TKA

Univariable logistic regression models showed that female sex, age 65–74 years, a higher WOMAC score, affliction duration of the impaired knee from 3 years to <6 years, and current use of analgesics were associated with higher PT utilization (Table 3).

Multivariable logistic regression analysis revealed that age 65–74 years, a higher WOMAC score, and current use of analgesics were associated with a higher probability of receiving PT. Furthermore, in that model, patients with a high educational level were associated with higher PT utilization (OR 3.13; 95% CI: 1.28 to 7.66), as were patients with no depressive symptoms. Female sex and affliction duration of the impaired knee from 3 years to <6 years did not remain statistically significant in the multivariable model. BMI and the number of comorbidities still had no influence on the utilization of PT.

## 4. Discussion

This study investigated PT utilization during the 12 months prior to TKA. The results suggest that only four out of 10 patients received physiotherapeutic treatment during this period. Increased PT utilization was associated with high educational level and higher disease burden. The presence of depressive symptoms and being 75+ years of age were associated with a lower probability of receiving PT.

Our finding that only 41% of patients received PT within 12 months prior to TKA is surprising, especially when taking into account that (1) guidelines recommend TKA only after a patient does not respond sufficiently to longer-term (at least 6 months of) non-surgical interventions [9,10] and (2) Skou et al. showed in a randomized trial that two-thirds of patients scheduled for TKA were able to delay their surgery for at least two years after participating in a 12-week comprehensive non-surgical treatment program [34]. However, our results are in line with the abovementioned German routine data analysis, which found that 49% of patients had utilized PT within 12 months prior TKA [17]. The small difference can be explained by the fact that that Lange et al. could not fully clarify PT indication or by regional disparities. Our prevalence result is higher than that found in a routine data analysis from the United States (14%) [16] but lower than found in a Canadian study (65%) [19] that assessed PT utilization 12 months prior to TKA. However, the TKA subgroup was rather small in the Canadian study (n = 34) and, thus, its results must be interpreted carefully. Higher PT utilization was also found in studies from the United Kingdom (49%) [20] and the Netherlands (73%) [21]. However, these studies only assessed “ever” use of PT prior to TKA, which hampers comparability. Overall, these data confirm findings from several studies, showing that the use of non-surgical measures in knee OA patients can be improved [35,36,37].

We found PT utilization to be associated with a higher WOMAC score as well as the current use of analgesics. This confirms results of previous studies that showed higher PT utilization with increased disease burden in individuals with rheumatoid arthritis (RA) [34], OA [38], and OA prior to TKA [19]. However, every second patient with severe functional limitation and pain (WOMAC score ≥59.0) still did not receive any PT in the twelve months prior to TKA. Although continuous PT management for years might not be possible, PT is probably underused, potentially reducing opportunities for pain relief, increased joint function, and therefore improved QoL [13,14]. The low utilization in individuals with high disease burden might be attributed to the fact that patients often perceive OA as a normal part of aging [19] or are not aware of the benefits of PT [39]. Furthermore, attitudes of the prescribing medical specialists towards PT for OA vary widely, leading to violations of guideline recommendations and related PT underutilization [37].

Although utilization of PT was generally low, we identified subgroups that received even less PT. As previously mentioned, age is discussed as an influencing factor for PT utilization, with inconsistent results being reported for OA populations and individuals prior to TKA [38,40,41]. For instance, Power et al. [19] observed no differences in patients one year prior to TKA, whereas King et al. [18] showed lower PT utilization in older TKA patients. We also found decreased utilization in the elderly (75+ years), which is consistent with findings from Yeh et al., who analyzed a large cohort with incident OA and observed significantly less PT utilization in older adults [41]. Similarly, Smith et al. found that older knee or hip OA patients received less PT and discussed that in the context of inequalities [42].

High income and higher education levels are associated with more frequent PT utilization in OA populations [38,40]. This was confirmed in our study as well as in two other studies on individuals prior to TKA [18,19]. As studies show that OA prevalence is higher among individuals with low SES [43] and that lower health literacy reduces the likelihood of receiving health services [44], these patients are doubly disadvantaged.

Studies on the association between psychological well-being and PT utilization in individuals with OA are scarce, although depression is a common comorbidity in pain patients [45]. King et al. observed higher PT utilization in patients with more depressive symptoms prior to TKA [18]. In contrast, we found lower odds of PT utilization in patients with moderate to severe depressive symptoms, a finding clearly calling for further research.

Female sex was associated with higher PT utilization in patients prior to TKA [17,18,19,22]. We also found a statistically significant association in the univariable logistic regression. However, this association did not remain significant in the multivariable analyses, potentially due to insufficient statistical power for this characteristic. Nevertheless, lower utilization in males was also observed in patients with OA [38,40,41].

### Strengths and Limitations

A major strength of the present study is the inclusion of patients immediately before surgery. As a result, patients were able to recall individual PT utilization prior to their TKA very well, which might reduce the potential for misclassification. Furthermore, this allowed clearly assigning PT to the impaired knee, which is not possible in routine data analyses. Moreover, unlike previous routine data analyses on the same topic [16,17], we were able to include important patient-reported outcomes and disease-related factors. Our 85% response rate is also noteworthy.

A few limitations must be considered. Due to the COVID-19 pandemic, recruitment had to be paused between 17 March and 13 May 2020 (recruitment stop 1) and between 18 December 2020 and 31 January 2021 (recruitment stop 2) because all elective surgeries were postponed. Therefore, we had to extend our scheduled recruitment period, which was originally planned for 12 months. Even more importantly, the COVID-19 pandemic might have influenced PT utilization. When stratifying by time periods, PT utilization was comparable before recruitment stops 1 and 2 (38% vs. 39%), while after recruitment stop 2, PT frequencies increased (49%). However, the sample size was small (n = 51) and the 95% CI rather wide (35.2–62.8).

Another aspect is that our study was limited to one university hospital, thus limiting the generalizability to other settings and regions. We were thus unable to evaluate regional differences in PT utilization that were shown elsewhere [17,40]. However, the hospital’s catchment area is large (only 31% of study participants resided in the city of the hospital), and as PT is most often prescribed by outpatient physicians in Germany [40], many different physicians from a larger area are involved. Therefore, our results do not reflect the prescribing behavior of only a few local physicians.

The final limitation is that patients might have received PT in the time before the 12 months prior to TKA. As therapists regularly establish home exercise programs with their clients, some patients might conceivably have already had such individualized programs in place. This would have led to underestimation of PT utilization in the year prior to surgery. In addition, patients might have been recommended treatment similar to PT (e.g., muscle strengthening in a gym) or received treatment by other healthcare professionals. However, among the participants who did not receive PT, only five reported having received treatment by chiropractors or osteopaths, suggesting that this factor had little influence on our findings.

## 5. Conclusions

We found little use of recommended PT management in patients prior to TKA. This potential underuse was particularly high in individuals 75+ years of age, those with low education levels, and patients with moderate-to-severe depressive symptoms. In addition, male sex has to be considered as a factor associated with underuse. Both prescribing medical specialists and patients should integrate PT more consistently into OA management. Furthermore, implementation strategies are required to optimize PT management of knee OA patients before progressing to TKA because doing so has the potential to delay or reduce the need for TKA, improve patient outcomes, and reduce healthcare costs.

## Figures and Tables

**Table 1:** Characteristics of the study population in %.

Characteristics	Female (n = 144) 59.8%	Male (n = 97) 40.2%	Total (n = 241) 100.0%
Age in years, mean (SD) (n = 241)	68.2 (9.6)	68.6 (9.1)	68.4 (9.4)
18–64 years	36.8	32.0	34.9
65–74 years	34.7	39.2	36.5
75+ years	28.5	28.9	28.6
Level of education (n = 237)			
low	50.0	50.5	50.2
middle	36.4	25.8	32.1
high	13.6	23.7	17.7
Living alone (n = 235)	29.6	12.9	23.0
General state of health (n = 238)			
(very) good	21.8	24.0	22.7
fair	49.3	40.6	45.8
(very) poor	28.9	35.4	31.5
BMI, mean (SD) (n = 241)	33.1 (6.8)	32.5 (5.1)	32.9 (6.2)
<25	8.3	6.2	7.5
25–<30	29.9	26.8	28.6
≥30	61.8	67.0	63.9
Elixhauser Comorbidity Index score [33] (n = 241)			
0	11.1	8.3	10.0
1–2	51.4	57.7	53.9
≥3	37.5	34.0	36.1
WOMAC score [24], mean (SD) (n = 230)	53.2 (14.0)	46.8 (14.7)	50.5 (14.6)
Q1	16.5	33.0	23.5
Q2	22.6	29.9	25.7
Q3	30.8	18.6	25.7
Q4	30.1	18.6	25.2
Duration of knee impairment in years, mean (SD) (n = 228)	7.0 (8.4)	8.3 (7.6)	7.6 (8.1)
<3 years	32.8	23.4	29.0
3–<6 years	32.1	33.0	32.5
≥6 years	35.1	43.6	38.6
Depressive symptoms (WHO-5) [29], mean (SD) (n = 233)	39.9 (24.3)	48.7 (25.2)	43.4 (25.0)
no	41.3	28.4	36.1
mild	23.9	22.1	23.2
moderate-to-severe	34.8	49.5	40.7
Use of analgesics, current (n = 233)	80.6	57.5	71.2

Values are presented as mean ± SD for continuous characteristics and as percentages otherwise. SD = standard deviation; SES = socioeconomic status; BMI = body mass index in kg/m^2^; WOMAC = Western Ontario and McMaster Universities Osteoarthritis Index [24]; Q1 = score <40.0; Q2 = score ≥40.0–<52.0; Q3 = score ≥52.0–<59.0; Q4 = score ≥59.0; WHO-5 = WHO-Five Well-Being Index [29].

**Table 2:** Utilization of physiotherapy (PT) in % with 95% confidence limits (CI), depending on covariates.

Characteristics	Proportion with PT
Overall (n = 239)	40.6 (34.4–46.8)
Sex (n = 239)	
female	48.3 (40.1–56.4)
male	29.2 (20.1–38.3)
Age groups (n = 239)	
18–64 years	36.1 (25.8–46.5)
65–74 years	51.1 (40.7–61.6)
75+ years	32.4 (21.2–43.5)
Level of education (n = 235)	
low	36.4 (27.8–45.1)
middle	41.3 (30.2–52.5)
high	50.0 (34.9–65.1)
Living alone (n = 234)	
yes	48.1 (34.8–61.5)
no	38.2 (31.0–45.2)
General state of health (n = 236)	
(very) good	44.4 (31.2–57.7)
fair	35.2 (26.2–44.2)
(very) poor	45.9 (34.6–57.3)
BMI (n = 239)	
<25	61.1 (38.6–83.6)
25–<30	38.2 (26.7–49.8)
≥30	39.2 (31.5–47.0)
Elixhauser Comorbidity Index score [33], (n = 239)	
0	54.2 (34.2–74.1)
1–2	39.1 (30.6–47.5)
≥3	39.1 (28.8–49.3)
WOMAC score [24], (n = 228)	
Q1	27.8 (15.8–39.7)
Q2	35.1 (22.7–47.5)
Q3	45.8 (33.1–58.5)
Q4	48.3 (35.4–61.1)
Duration of knee impairment in years (n = 227)	
<3 years	36.9 (25.2–48.7)
3–<6 years	50.0 (38.6–61.4)
≥6 years	34.1 (24.2–44.0)
Depressive symptoms (WHO-5) [29], (n = 231)	
no	41.9 (31.9–52.0)
mild	42.6 (29.4–55.8)
moderate-to-severe	38.1 (27.7–48.5)
Use of analgesics (n = 233)	
yes	45.8 (38.2–53.4)
no	25.4 (15.0–35.8)

SES = socioeconomic status; BMI = body mass index in kg/m^2^; WOMAC = Western Ontario and McMaster Universities Osteoarthritis Index [24]; Q1 = score <40.0; Q2 = score ≥40.0–<52.0; Q3 = score ≥52.0–<59.0; Q4 = score ≥59.0; WHO-5 = WHO-Five Well-Being Index [29].

**Table 3:** Factors associated with a higher utilization of physiotherapy (PT) prior to TKA: results from univariable and multivariable logistic regression analyses (n = 206).

Characteristics	Reference	OR (95% CI)	
		Univariable Analysis	Multivariable Analysis
Sex			
female	male	**2.26 (1.31–3.92)**	1.74 (0.88–3.42)
Age group			
18–64 years	75+ years	1.18 (0.60–2.33)	0.89 (0.37–2.16)
65–74 years	75+ years	**2.19 (1.13–4.23)**	**3.41 (1.41–8.25)**
Level of education			
middle	low	1.23 (0.68–2.22)	1.28 (0.60–2.70)
high	low	1.74 (0.86–3.55)	**3.13 (1.28–7.66)**
BMI			
<25	≥30	2.44 (0.90–6.63)	2.71 (0.72–10.23)
25–<30	≥30	0.96 (0.53–1.73)	0.77 (0.34–1.77)
Elixhauser Comorbidity Index score [33]			
1–2	0	0.54 (0.23–1.31)	0.54 (0.18–1.62)
≥3	0	0.54 (0.22–1.35)	0.43 (0.13–1.44)
WOMAC score [24]			
Q2	Q1	1.41 (0.63–3.15)	1.90 (0.68–5.34)
Q3	Q1	**2.19 (1.00–4.81)**	**3.50 (1.15–10.69)**
Q4	Q1	**2.43 (1.10–5.33)**	**4.65 (1.46–14.78)**
Duration of knee impairment in years			
<3 years	≥6 years	1.13 (0.58–2.21)	1.66 (0.74–3.75)
3–<6 years	≥6 years	**1.93 (1.03–3.64)**	1.90 (0.89–4.08)
Depressive symptoms (WHO-5) [29]			
mild	no	1.03 (0.52–2.03)	0.60 (0.24–1.47)
moderate-to-severe	no	0.85 (0.47–1.56)	**0.38 (0.16–0.92)**
Use of analgesics			
yes	no	**2.48 (1.32–4.66)**	**3.35 (1.44–7.76)**

Odds ratios of variables significantly associated with the utilization of physiotherapy are shown in bold. TKA = total knee arthroplasty; OR = odds ratio; CI = confidence limit; SES = socioeconomic status; BMI = body mass index in kg/m^2^; WOMAC = Western Ontario and McMaster Universities Osteoarthritis Index [24]; Q1 = score <40.0; Q2 = score ≥40.0–<52.0; Q3 = score ≥52.0–<59.0; Q4 = score ≥59.0; WHO-5 = WHO-Five Well-Being Index [29].

## Data Availability

The datasets generated during and/or analyzed during the current study are available from the corresponding author on reasonable request.

## Supplementary Materials

The following supporting information can be downloaded at: https://www.mdpi.com/article/10.3390/healthcare10020407/s1, Supplementary Material S1: Utilization of physiotherapy in % with 95% confidence limits, stratified by sex; Supplementary Material S2: Mean number of physiotherapeutic treatments/sessions with standard deviation and interquartile range, stratified by sex.

## References

[1] Woolf A.D., Pfleger B. (2003). Burden of major musculoskeletal conditions. Bull. World Health Organ..

[2] Martel-Pelletier J., Barr A., Cicuttini F., Conaghan P., Cooper C., Goldring M.B., Goldring S.R., Jones G., Teichtahl A.J., Pelletier J.-P. (2016). Osteoarthritis. Nat. Rev. Dis. Prim..

[3] Cudejko T., Van Der Esch M., Van Der Leeden M., Holla J., Roorda L.D., Lems W., Dekker J. (2018). Proprioception mediates the association between systemic inflamma-tion and muscle weakness in patients with knee osteoarthritis: Results from the Amsterdam Osteoarthritis cohort. J. Rehabil. Med..

[4] Osaki M., Tomita M., Abe Y., Ye Z., Honda S., Yoshida S., Shindo H., Aoyagi K. (2012). Physical performance and knee osteoarthritis among community-dwelling women in Japan: The Hizen-Oshima Study, cross-sectional study. Rheumatol. Int..

[5] Cross M., Smith E., Hoy D., Nolte S., Ackerman I., Fransen M., Bridgett L., Williams S., Guillemin F., Hill C.L. (2014). The global burden of hip and knee osteoarthritis: Estimates from the Global Burden of Disease 2010 study. Ann. Rheum. Dis..

[6] Ethgen O., Bruyère O., Richy F., Dardennes C., Reginster J.-Y. (2004). Health-Related Quality of Life in Total Hip and Total Knee Arthroplasty: A qualitative and systematic review of the literature. J. Bone Jt. Surg. Am..

[7] Beswick A.D., Wylde V., Gooberman-Hill R., Blom A.W., Dieppe P. (2012). What proportion of patients report long-term pain after total hip or knee replacement for osteoarthritis? A systematic review of prospective studies in unselected patients. BMJ Open.

[8] Glyn-Jones S., Palmer A.J.R., Agricola R., Price A.J., Vincent T., Weinans H., Carr A.J. (2015). Osteoarthritis. Lancet.

[9] Hochberg M.C., Altman R.D., April K.T., Benkhalti M., Guyatt G., McGowan J., Towheed T., Welch V., Wells G., Tugwell P. (2012). American College of Rheumatology 2012 recommendations for the use of nonpharmacologic and pharmacologic therapies in osteoarthritis of the hand, hip, and knee. Arthritis Care Res..

[10] Nelson A.E., Allen K.D., Golightly Y., Goode A.P., Jordan J.M. (2014). A systematic review of recommendations and guidelines for the management of osteoarthritis: The Chronic Osteoarthritis Management Initiative of the U.S. Bone and Joint Initiative. Semin. Arthritis Rheum..

[11] Hunter D.J., Bierma-Zeinstra S. (2019). Osteoarthritis. Lancet.

[12] Roos E., Juhl C. (2012). Osteoarthritis 2012 year in review: Rehabilitation and outcomes. Osteoarthr. Cartil..

[13] Fransen M., McConnell S., Harmer A.R., Van Der Esch M., Simic M., Bennell K. (2015). Exercise for osteoarthritis of the knee. Cochrane Database Syst. Rev..

[14] A Uthman O., A van der Windt D., Jordan J., Dziedzic K.S., Healey E.L., Peat G.M., Foster N.E. (2014). Exercise for lower limb osteoarthritis: Systematic review incorporating trial sequential analysis and network meta-analysis. Br. J. Sports Med..

[15] Zhang W., Nuki G., Moskowitz R., Abramson S., Altman R., Arden N., Bierma-Zeinstra S., Brandt K., Croft P., Doherty M. (2010). OARSI recommendations for the management of hip and knee osteoarthritis: Part III: Changes in evidence following systematic cumulative update of research published through January 2009. Osteoarthr. Cartil..

[16] Bedard N.A., Dowdle S.B., Anthony C.A., DeMik D.E., McHugh M.A., Bozic K.J., Callaghan J.J. (2017). The AAHKS Clinical Research Award: What Are the Costs of Knee Osteoarthritis in the Year Prior to Total Knee Arthroplasty?. J. Arthroplast..

[17] Lange T., Ramos A.L., Albrecht K., Günther K.-P., Jacobs H., Schmitt J., Hoffmann F., Goronzy J., Postler A. (2018). Verordnungshäufigkeit physikalischer Therapien und Analgetika vor dem Einsatz einer Hüft- bzw. Kniegelenks-Endoprothese. Der Orthopäde.

[18] King L.K., Marshall D.A., Faris P., Woodhouse L.J., Jones C.A., Noseworthy T., Bohm E., Dunbar M.J., Hawker G.A. (2019). Use of Recommended Non-surgical Knee Osteoarthritis Management in Patients prior to Total Knee Arthroplasty: A Cross-sectional Study. J. Rheumatol..

[19] Power J.D., A Cott C., Badley E.M., A Hawker G. (2005). Physical therapy services for older adults with at least moderately severe hip or knee arthritis in 2 Ontario counties. J. Rheumatol..

[20] McHugh G.A., Luker K., Campbell M., Kay P.R., Silman A.J. (2006). A longitudinal study exploring pain control, treatment and service provision for individuals with end-stage lower limb osteoarthritis. Rheumatology.

[21] Hofstede S.N., Vlieland T.P.M.V., Ende C.H.M.V.D., Nelissen R.G.H.H., de Mheen P.J.M.-V., van Bodegom-Vos L. (2015). Variation in use of non-surgical treatments among osteoarthritis patients in orthopaedic practice in the Netherlands. BMJ Open.

[22] Bawa H.S., Weick J.W., Dirschl D.R. (2016). Gender Disparities in Osteoarthritis-Related Health Care Utilization before Total Knee Arthroplasty. J. Arthroplast..

[23] Gunther K.P., Sturmer T., Sauerland S., Zeissig I., Sun Y., Kessler S., Scharf H.P., Brenner H., Puhl W. (1998). Prevalence of generalised osteoarthritis in patients with advanced hip and knee osteoarthritis: The Ulm Osteoarthritis Study. Ann. Rheum. Dis..

[24] Bellamy N. (2005). The WOMAC Knee and Hip Osteoarthritis Indices: Development, validation, globalization and influence on the development of the AUSCAN Hand Osteoarthritis Indices. Clin. Exp. Rheumatol..

[25] Escobar A., Quintana J.M., Bilbao A., Arostegui I., Lafuente I., Vidaurreta I. (2007). Responsiveness and clinically important differences for the WOMAC and SF-36 after total knee replacement. Osteoarthr. Cartil..

[26] Picavet H.S.J. (2004). Health related quality of life in multiple musculoskeletal diseases: SF-36 and EQ-5D in the DMC3 study. Ann. Rheum. Dis..

[27] Scheidt-Nave C., Kamtsiuris P., Gößwald A., Hölling H., Lange M., A Busch M., Dahm S., Dölle R., Ellert U., Fuchs J. (2012). German health interview and examination survey for adults (DEGS)—Design, objectives and implementation of the first data collection wave. BMC Public Health.

[28] Ellert U., Kurth B.-M. (2004). Methodische Betrachtungen zu den Summenscores des SF-36 anhand der erwachsenen bundesdeutschen Bevölkerung. Bundesgesundheitsblatt-Gesundheitsforschung-Gesundheitsschutz.

[29] Topp C.W., Østergaard S.D., Søndergaard S., Bech P. (2015). The WHO-5 Well-Being Index: A Systematic Review of the Literature. Psychother. Psychosom..

[30] Boonstra A.M., Preuper H.R.S., Balk G.A., Stewart R.E. (2014). Cut-off points for mild, moderate, and severe pain on the visual analogue scale for pain in patients with chronic musculoskeletal pain. Pain.

[31] UNESCO (1997). International Standard Classification of Education ISCED 1997.

[32] UNESCO (2011). International Standard Classification of Education ISCED 2011.

[33] Elixhauser A., Steiner C., Harris D.R., Coffey R.M. (1998). Comorbidity Measures for Use with Administrative Data. Med. Care.

[34] Skou S.T., Roos E., Laursen M., Rathleff M.S., Arendt-Nielsen L., Rasmussen S., Simonsen O. (2018). Total knee replacement and non-surgical treatment of knee osteoarthritis: 2-year outcome from two parallel randomized controlled trials. Osteoarthr. Cartil..

[35] Li L.C., Maetzel A., Pencharz J.N., Maguire L., Bombardier C. (2004). The Community Hypertension and Arthritis Project (CHAP) Team Use of mainstream nonpharmacologic treatment by patients with arthritis. Arthritis Care Res..

[36] Brand C.A., Harrison C., Tropea J., Hinman R.S., Britt H., Bennell K. (2014). Management of Osteoarthritis in General Practice in Australia. Arthritis Care Res..

[37] Cottrell E., Roddy E., Foster N.E. (2010). The attitudes, beliefs and behaviours of GPs regarding exercise for chronic knee pain: A systematic review. BMC Fam. Pract..

[38] Iversen M.D., A Schwartz T., von Heideken J., Callahan L., Golightly Y., Goode A., Hill C., Huffman K., Pathak A., Cooke J. (2018). Sociodemographic and Clinical Correlates of Physical Therapy Utilization in Adults With Symptomatic Knee Osteoarthritis. Phys. Ther..

[39] Hsieh J.B., Dominick K.L. (2003). Use of non-pharmacological therapies among patients with osteoarthritis. Aging Clin. Exp. Res..

[40] Jacobs H., Callhoff J., Albrecht K., Postler A., Saam J., Lange T., Goronzy J., Günther K.-P., Hoffmann F. (2021). Use of Physical Therapy in Patients with Osteoarthritis in Germany: An Analysis of a Linkage of Claims and Survey Data. Arthritis Care Res..

[41] Yeh H.-J., Chou Y.-J., Yang N.-P., Huang N. (2015). Receipt of Physical Therapy among Osteoarthritis Patients and Its Influencing Factors. Arch. Phys. Med. Rehabil..

[42] Smith T., Collier T.S., Smith B., Mansfield M. (2019). Who seeks physiotherapy or exercise treatment for hip and knee osteoarthritis? A cross-sectional analysis of the English Longitudinal Study of Ageing. Int. J. Rheum. Dis..

[43] A A Dalstra J., E Kunst A., Borrell C., Breeze E., Cambois E., Costa G., Geurts J.J.M., Lahelma E., Van Oyen H., Rasmussen N.K. (2005). Socioeconomic differences in the prevalence of common chronic diseases: An overview of eight European countries. Int. J. Epidemiol..

[44] Rademakers J., Heijmans M. (2018). Beyond Reading and Understanding: Health Literacy as the Capacity to Act. Int. J. Environ. Res. Public Health.

[45] Redeker I., Hoffmann F., Callhoff J., Haibel H., Sieper J., Zink A., Poddubnyy D. (2018). Determinants of psychological well-being in axial spondyloarthritis: An analysis based on linked claims and patient-reported survey data. Ann. Rheum. Dis..

