# Supplementary material for: Use of Physiotherapy Prior to Total Knee Arthroplasty—Results of the Prospective FInGK Study
Source: Healthcare (Basel). 2022 Feb 21;10(2):407. doi: 10.3390/healthcare10020407 (PMC8871805; doi:10.3390/healthcare10020407)
Supplement: Supplementary file 1 [file healthcare-10-00407-s001.zip › healthcare-1580455-supplementary.pdf]

Supplementary material

Supplementary material S1: Utilization of physiotherapy (PT) in % with 95% confidence limits (CI), stratified by sex

	Female (n=143)	Male (n=96)	Total (n=239)
**At least one PT**	48.3 (40.1-56.4)	29.2 (20.1-38.3)	40.6 (34.4-46.8)
**Therapeutic exercises, massage therapy, manual therapy, traction therapy**	46.2 (38.0-54.3)	25.9 (16.3-33.7)	37.7 (31.5-43.8)
**Manual lymphatic drainage**	14.0 (8.3-19.7)	5.2 (0.8-9.7)	10.5 (6.6-14.3)
**Electrotherapy**	4.9 (1.4-8.4)	2.1 (0.0-4.9)	3.8 (1.4-6.2)
**Thermotherapy**	3.5 (0.5-6.5)	-	2.1 (0.3-3.9)
**Other**	2.8 (0.1-5.5)	1.0 (0.0-3.1)	2.1 (0.3-3.9)

Supplementary material S2: Mean number of physiotherapeutic treatments/sessions with standard deviation and interquartile range, stratified by sex

		Female (n=143)	Male (n=96)	Total (n=239)
**At least one PT**	Mean (SD)	31.4 (36.6)	25.5 (33.2)	29.6 (35.3)
	IQR	(12-36)	(10-30)	(12-30)
**Therapeutic exercises, massage therapy, manual therapy, traction therapy**	Mean (SD)	26.9 (30.3)	20.5 (19.2)	25.1 (27.7)
	IQR	(10-30)	(9.0-24)	(10-25)
**Manual lymphatic drainage**	Mean (SD)	17.9 (17.0)	38.5 (36.8)	21.7 (22.2)
	IQR	(6-30)	(12-65)	(6-30)
**Electrotherapy**	Mean (SD)	5.7 (2.7)	16.0 (5.7)	8.3 (5.7)
	IQR	(4-6)	(12-20)	(5-10)
**Thermotherapy**	Mean (SD)	6.0 (0)	-	6.0 (0)
	IQR	(6-6)		(6-6)
**Other**	Mean (SD)	5.3 (5.0)	30.0 (0)	10.2 (11.9)
	IQR	(1.5-9.0)	(30-30)	(2-12)

PT=physiotherapy; SD=standard deviation; IQR=interquartile range

Treatments/sessions were determined by asking; “Did you receive PT in the last 12 months due to the impaired knee joint? How many treatments/sessions did you have? (For information: One physiotherapy prescription usually comprises 6 treatments/sessions of 20-30 minutes each).”
